# Pellagra Preventable

**Published:** 1916-05

**Authors:** 


### Pellagra Preventable.

It has about been demonstrated that pellagra is preventable and that it is a dietary instead of a contagious disease. This being true, every Southern housewife should take some kind of a course that will enable her to intelligently arrange the family meals so that the family may keep in good health. This is true whether there is fear of pellagra or not. Unquestionably much disease is due to lack of proper nourishment. Many families that have enough food and food that is expensive enough suffer from a lack of knowledge as to what constitutes a properly balanced ration. Schools of domestic economy are doing something to teach housewives how to feed their families, but they reach comparativley few people. It is going to be up to the family physician and the newspapers to help spread this knowledge. If pellagra can be prevented by proper feeding, ignorance of what is proper dieting is almost criminal; at any rate it is inexcusable in this country where climatic conditions seem to combine with improper diet in spreading the disease.

